# DNA barcoding data release for Coleoptera from the Gunung Halimun canopy fogging workpackage of the Indonesian Biodiversity Information System (IndoBioSys) project

**DOI:** 10.3897/BDJ.7.e31432

**Published:** 2019-01-15

**Authors:** Bruno Cancian de Araujo, Stefan Schmidt, Olga Schmidt, Thomas von Rintelen, Kristina von Rintelen, Andreas Floren, Rosichon Ubaidillah, Djunijanti Peggie, Michael Balke

**Affiliations:** 1 SNSB-Zoologische Staatssammlung München, Munich, Germany SNSB-Zoologische Staatssammlung München Munich Germany; 2 Museum für Naturkunde, Berlin, Germany Museum für Naturkunde Berlin Germany; 3 The Biocenter of the University of Würzburg, Würzburg, Germany The Biocenter of the University of Würzburg Würzburg Germany; 4 Museum Zoologicum Bogoriense, Research Center for Biology, Indonesian Institute of Sciences, Cibinong, Indonesia Museum Zoologicum Bogoriense, Research Center for Biology, Indonesian Institute of Sciences Cibinong Indonesia

**Keywords:** Beetles, BIN, biodiversity, BOLD, Cikaniki, collecting methods, databasing, DNA barcoding, Gunung Botol, Insecta, inventory, Java, monitoring

## Abstract

We present the results of a DNA barcoding pipeline that was established as part of the German-Indonesian IndobioSys project - Indonesian Biodiversity Information System. Our data release provides the first large-scale diversity assessment of Indonesian coleoptera obtained by canopy fogging. The project combined extensive fieldwork with databasing, DNA barcode based species delineation and the release of results in collaboration with Indonesian counterparts, aimed at supporting further analyses of the data. Canopy fogging on 28 trees was undertaken at two different sites, Cikaniki and Gunung Botol, in the south-eastern area of the Gunung Halimun-Salak National Park in West Java, Indonesia. In total, 7,447 specimens of Coleoptera were processed, of which 3,836 specimens produced DNA barcode sequences that were longer than 300 bp. A total of 3,750 specimens were assigned a Barcode Index Number (BIN), including 2,013 specimens from Cikaniki and 1,737 specimens from Gunung Botol. The 747 BINs, that were obtained, represented 39 families of Coleoptera. The distribution of specimens with BINs per tree was quite heterogeneous in both sites even in terms of the abundance of specimens or diversity of BINs. The specimen distribution per taxon was heterogeneous as well. Some 416 specimens could not be identified to family level, corresponding to 72 BINs that lack a family level identification. The data have shown a large heterogeneity in terms of abundance and distribution of BINs between sites, trees and families of Coleoptera. From the total of 747 BINs that were recovered, 421 (56%) are exclusive from a single tree. Although the two study sites were in close proximity and separated by a distance of only about five kilometres, the number of shared BINs between sites is low, with 81 of the 747 BINs. With this data release, we expect to shed some light on the largely hidden diversity in the canopy of tropical forests in Indonesia and elsewhere.

## Introduction

Insects and other invertebrates account for over 97% of multicellular animal species diversity (Groombridge 1992) and their predominance stresses the importance of incorporating invertebrate data into studies on biodiversity, ecology and conservation (e.g. Myers et al. 2000, Myers and Mittermeier 2003). At the same time, the high species diversity in tropical regions (Groombridge 1992), the need for processing samples with large numbers of individuals and high levels of endemism often prevent them from being incorporated into biodiversity related research projects, leading to a strong taxonomic bias in biodiversity data (Troudet et al. 2017). Sorting samples with thousands of specimens and their identification to species level is challenging and often virtually impossible, but it is the basis for downstream applications. Traditional methods using morphological approaches suffer from lack of taxonomic specialists (Wheeler and Cracraft 1996, Wheeler 2004). Recent advances in molecular biodiversity assessment employing DNA barcoding allow the discovery and characterisation of large numbers of specimens in a short timeframe. DNA barcoding sets out to overcome the problem of a fast and reliable biodiversity assessment (e.g. Hebert et al. 2003, Janzen et al. 2009). The method has proven to be exceedingly efficient and useful as a tool for both the reliable and fast identification of animals and plants and for the discovery of new species. The method allows the characterisation of a large numbers of specimens and species with high accuracy in a short amount of time and, most importantly, provides the data that are needed for ecological research, monitoring, conservation and biodiversity studies.

**Figure 1.**
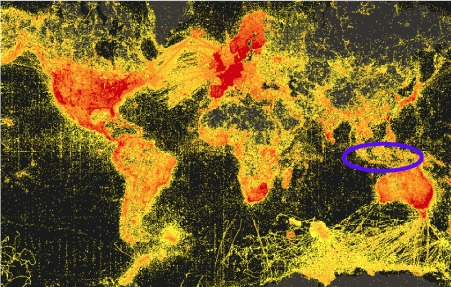
Heat map showing the worldwide distribution of occurrence records provided by the Global Biodiversity Information Facility (GBIF, www.gbif.org, accessed on 15-Dec-2018). Only 0.19% of the approximately one billion occurrence records that are accessible through GBIF are from Indonesia (blue ellipse), despite Indonesia's being amongst the top global biodiversity hotspots.

Here we focus on Indonesia, one of the most biodiversity rich countries on Earth, with its diversity, however, largely undiscovered and not described scientifically (Fig. 1).

The Indonesian archipelago comprises two of the world’s biodiversity hotspots, i.e. areas with a high degree of endemic species that are highly threatened by loss of habitats (see Mittermeier et al. 1997, Myers et al. 2000, Bruyn et al. 2014, Ceballos et al. 2015). Its insular character and complex geological history led to the evolution of megadiverse fauna and flora on the global scale (Lohman et al. 2011, Rintelen et al. 2017). The challenges that the taxonomic inventory of the local fauna faces is summarised by Cancian de Araujo (in prep.).

To accelerate the biodiversity discovery process, as well as to increase our knowledge about the archipelago fauna, the German-Indonesian IndoBioSys project (IndoBioSys, Indonesian Biodiversity Discovery and Information System) combines species discovery and species characterisation using morphology and DNA sequence data (e.g. Rintelen et al. 2017, Cancian de Araujo et al. 2018, Cancian de Araujo et al. 2017, Schmidt et al. 2017). The major aim of this still ongoing project is to develop a standardised and sustainable biodiversity discovery workflow to accelerate the process of species discovery from underexplored biodiversity-rich areas like Indonesia. This primarily includes biodiversity data (e.g. from sampling, identification, imaging and storing of specimens), but also information on promising target taxa with a high potential for biotechnological research (such as for the discovery of new anti-infective compounds: functional screening approach). A preliminary project summary can be found in Rintelen (2018).

Here, we present the results of a DNA barcoding pipeline to provide the first objective diversity assessment of Indonesian forest canopy Coleoptera. This is based on the canopy fogging workpackage of the German-Indonesian IndoBioSys project (IndoBioSys) that combined extensive fieldwork in the Gunung Halimun-Salak National Park in West Java with databasing, molecular species delineation and joint release of results in support of further analysis. A project outline was provided by Cancian de Araujo et al. (2017) and the working principle was further elaborated by Cancian de Araujo et al. (2018).

## Methods

Data are stored in and were analysed with the Barcode of Life Data Systems (BOLD). A summary of fieldwork and laboratory procedures related to this study are given in Cancian de Araujo et al. (2017) and Schmidt et al. (2017). Methodological steps specific to the work package presented here are described below.

### Fieldwork and processing of samples

**Figure 2.**
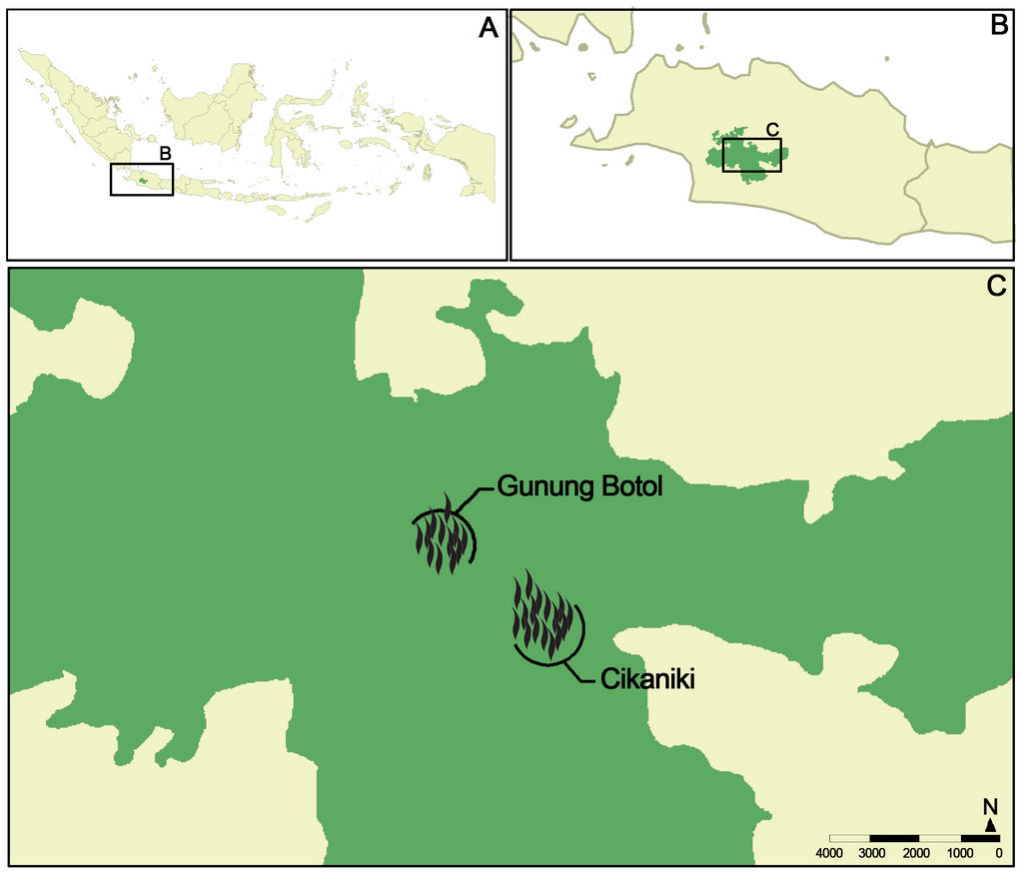
Collecting sites in the Gunung Halimun-Salak National Park.

Canopy fogging was performed at two different sites, Cikaniki and Gunung Botol, in the south-eastern part of the Gunung Halimun-Salak National Park in West Java, Indonesia (Fig. 2). Both sites are separated from each other by about five kilometres of continuous forest and by a difference in altitude of about 600 metres, with Cikaniki at 1,100 m a.s.l. and Gunung Botol at 1,700 m a.s.l. Altogether, 28 trees were sampled (Table 1), 17 at Cikaniki and 11 at Gunung Botol.

**Figure 3.**
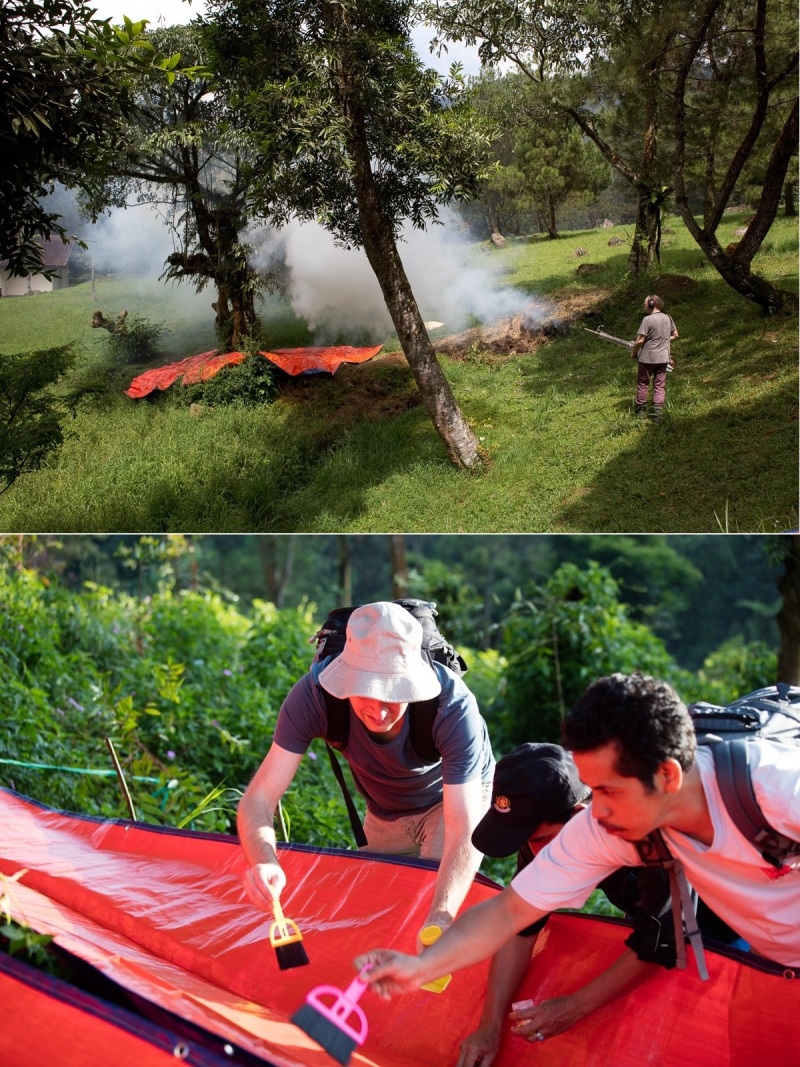
Insecticidal fogging technique (images by B. Schurian, MfN Berlin).

Specimens were collected using an insecticidal fogging technique (Floren 2010). Collecting sheets were placed on the ground under the trees (Fig. 3). As insecticide, a natural pyrethrum was used that degrades into non-toxic components within a few hours after exposure to light. About two hours after the fogging event, insects were transferred to 96% ethanol using brushes and stored at room temperature in the lab for further processing.

The samples were processed at the ZSM in Munich, Germany where they were sorted to ordinal level. All Coleoptera that were used for molecular biodiversity assessment were mounted on card labels and labelled. All specimens will be repatriated to MZB at the end of the project. This research was conducted under the foreign research permit granted by the Ministry of Research and Higher Education of the Republic of Indonesia number 2B/TKPIPA/E5/Dit.KI/II/2016.

### Terminology

The terms used to refer to BINs abundance and diversity when presenting and discussing the data are as follows. The term "specimen with sequence" refers to specimens processed that had sequences with a length of least 300 bp, "specimen with BIN" refers to specimens processed that were assigned a BIN by the BOLD system and "exclusive BINs" refers to the number of BINs (pre-existing and new to BOLD) that are unique for a specific site, tree or taxon, in other words, the BINs that are not shared with any other site, tree or taxon.

### Data analysis

Collection and molecular data were downloaded from the BOLD IndoBioSys campaign projects. The records recovered were filtered by insect order and collecting method, those being the records corresponding to "Coleoptera" collected by "fogging", individualised in separate Excel worksheets for data release and descriptive analysis of spatial and diversity distribution.

All specimen data are accessible in BOLD as a single citable dataset (dx.doi.org/10.5883/DS-INFOGCOL). The data include the record identifier, collecting locality, geographic coordinates, elevation, collector and specimen image. Sequence data can be obtained through BOLD and include a detailed LIMS report, primer informationand access to trace files. The sequences are also available on GenBank (accession numbers MK080571-MK084473).

### Specimens identification

Most specimens were identified at the family level before submission to the molecular analysis pipeline and to the BOLD platform. After BIN assignment by the BOLD system, specimens that were not identified at the family level and which succeed in receiving a BIN were identified by using the BOLD identification system (BOLD taxonomy match). The BIN-based identification was double-checked by comparing images with images of previously identified specimens and, if necessary, using a tree-based identification approach (NJ-trees).

## Results

**Figure 4.**
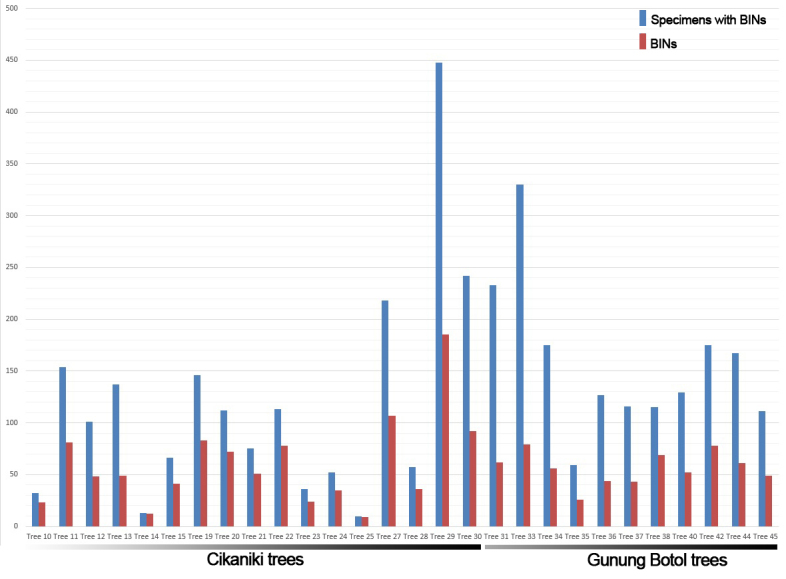
BIN distribution per site and tree.

In total, 7,447 specimens of Coleoptera were processed. From those, 3,836 specimens produced CO1-5P sequences longer than 300 base pairs, representing a success rate of 50.35%, with 3,750 of those specimens receiving a BIN. In total, it corresponded to 747 BINs, distributed heterogeneously over 39 families of Coleoptera. The total amount of specimens with BINs was evenly distributed between the two sites being 2,013 specimens (53.68%) for Cikaniki and 1,737 specimens (46.32%) for Gunung Botol. Despite that, the diversity of BINs found in both sites was not as even as that, with 557 BINs found in Cikaniki and 271 BINs found in Gunung Botol corresponding, respectively, to 67.27% and 32.73% of the BINs diversity. The distribution of specimens with BINs per tree was quite heterogeneous in both sites (especially in Cikaniki) even in terms of the abundance of specimens or diversity of BINs. In terms of abundance, tree 25 from Cikaniki contributed only 10 specimens with BINs when tree 29, at the same site, contributed 448 specimens with BINs, a difference of 44.8 times (Fig. 4). For Gunung Botol, the tree contribution was more even with a difference of 5.59 times between the tree that contributed less (tree 35, 59 specimens with BINs) and the tree that has contributed more (tree 33, 330 specimens with BINs). In terms of diversity, the largest difference was in Cikaniki with tree 29 contributing 20.5 times more BINs than tree 25 (185 and 9 BINs, respectively). The BIN sharing between the two sites was low with only 81 BINs shared, meaning that 429 BINs were found exclusively at Cikaniki and 190 BINs were found exclusively at Gunung Botol.

**Figure 5.**
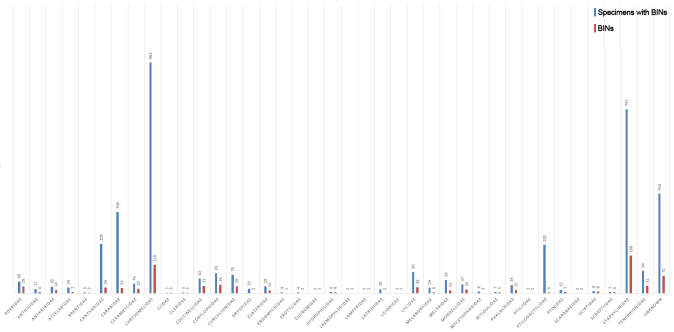
BIN distribution per family

**Figure 6.**
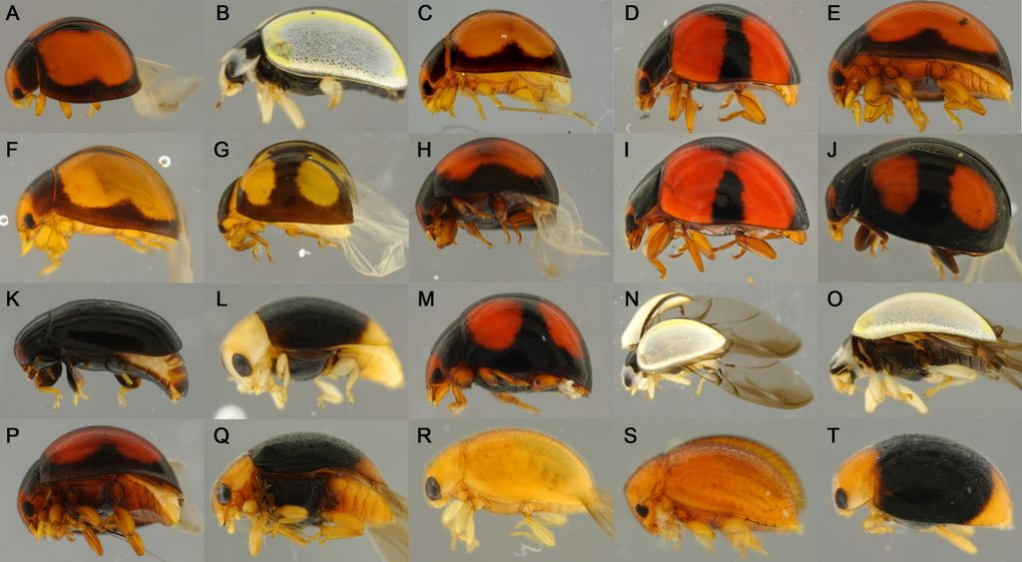
Part of the Coccinellidae morphological diversity and its correspondent BINs: **A.** BOLD:ADG7156, **B.** BOLD:ADG0348 **C.** BOLD:ADG7156 **D.** BOLD:ADH1869 **E.** BOLD:ADG7156 **F.** BOLD:ADG7156 **G.** BOLD:ADG3219 **H.** BOLD:ADG3219 **I.** BOLD:ADH0801 **J.** BOLD:ADG3219 **K.** BOLD:ADC1473 **L.** BOLD:ADC1743 **M.** BOLD:ADG8109 **N.** BOLD:ADG0348 **O.** BOLD:ADG0348 **P.** BOLD:ADG7156 **Q.** BOLD:ADA5666 **R.** BOLD:ADD3008 **S.** BOLD:ADH3343 **T.** BOLD:ADG0264.

The specimen distribution per taxon was heterogeneous as well, with only one family (Chrysomelidae) revealing 961 specimens with BINs, more than a quarter of the total found. The two families with most specimens (Chrysomelidae and Staphylinidae) contributed more to the total of specimens with BINs than all other families together with the 25 families with fewer specimens contributing with less than 1% of the total each (Fig. 5). A total of 416 specimens were not able to be identified to family level corresponding to 72 BINs with no family identification. In terms of diversity, the discrepancy between the families was lower, indicating a more homogenous distribution of BINs per family. Some families as Ptilodactylidae that substantially contributed with the total amount of specimens (202 specimens, 5.4% of the total) did not contribute in the same way for the total diversity (7 BINs, 0.93% of the total). On the other hand, Coccinellidae contributed only 1.7% of the total specimens but 4.1% of the total diversity (Fig. 6).

## Discussion

The data show a large heterogeneity in terms of abundance and distribution of BINs across sites, trees and families of Coleoptera. From the total of 747 BINs found, 421 (56.4%) are exclusive to a single tree. The low amount of BINs shared between Cikaniki and Gunung Botol sites (81 of the 747 BINs) is impressive considering that those sites are separated by only five kilometres of continuous forest. This might suggest a high altitudinal stratification since Cikaniki is on average 600 metres lower than Gunung Botol. This diversity is also not evenly distributed per tree, with the exclusivity of BINs per tree varying between 2.3% to 20.4% of the total of BINs. Tree 29 from Cikaniki site exemplifies the complex distribution of the Coleoptera diversity over this area. Despite being the tree with the largest contribution in terms of specimens and sequences (972 specimens, 448 producing BINs), most of those specimens share the same BIN ending in a diversity of only 185 BINs for this tree, which indicates an ambient with high abundance and low diversity of beetles. Despite that, 74 of those 185 BINs (16.5%) were found exclusively at this tree, showing that this relatively low diversity is potentially endemic.

**Figure 7.**
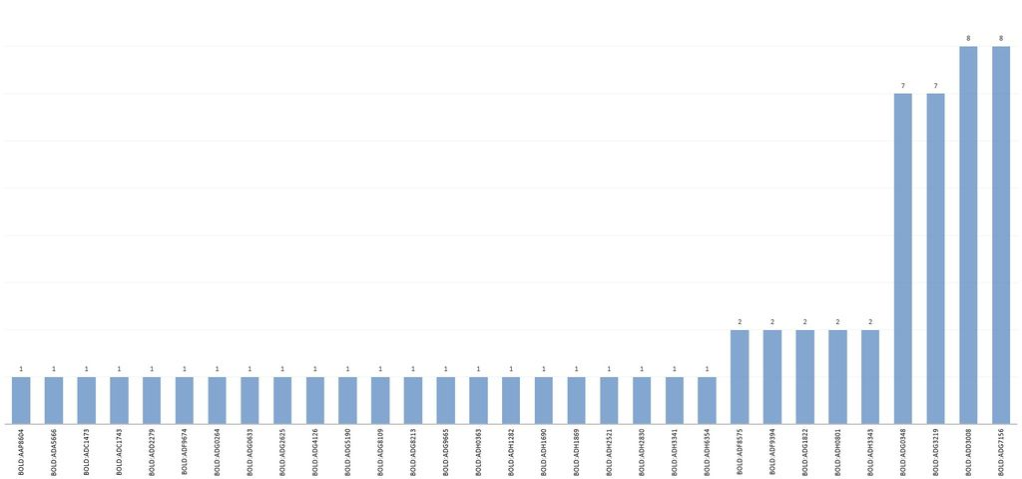
Coccinellidae specimens distribution per BIN.

**Figure 8.**
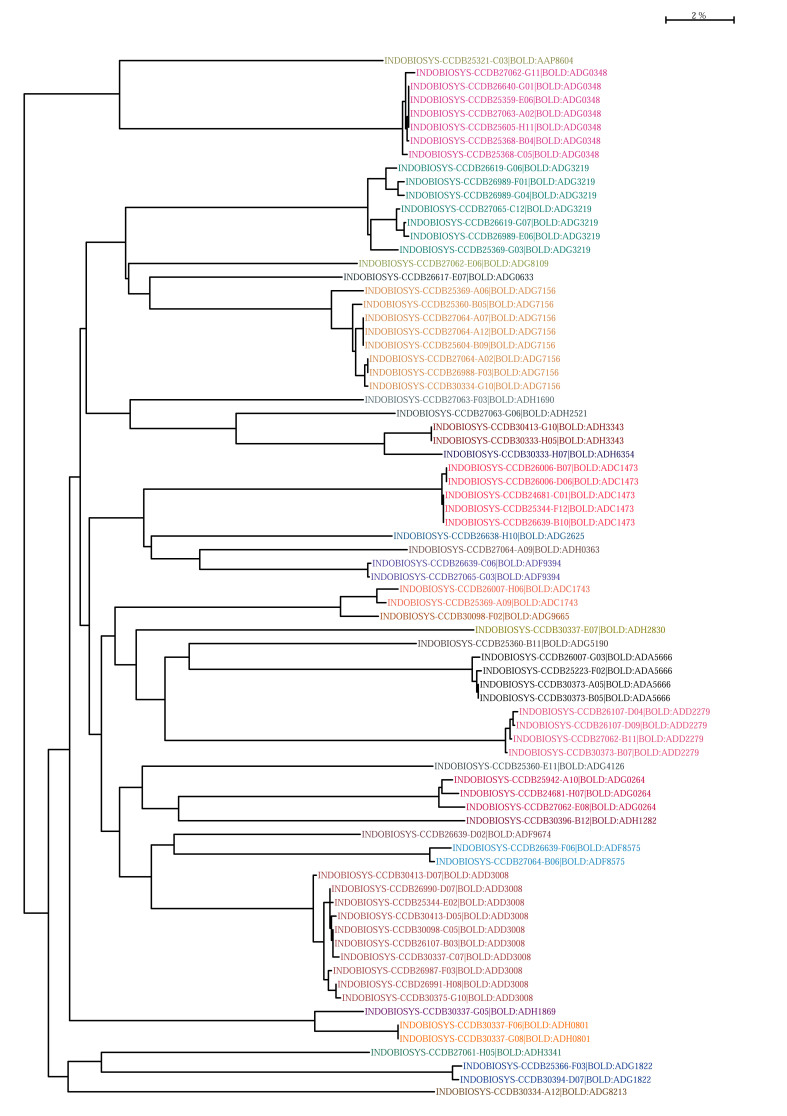
Neighbour joining tree (K2P distance model) of Coccinellidae specimens with respective BOLD sample IDs and BINs. The BIN clusters are represented by different colours.

At the same time, abundance and diversity were not necessarily related when analysing the families of Coleoptera. The family Coccinellidae has shown to be both diverse and abundant (Fig. 7) with 62 specimens in total representing 31 BINs or potential putative species (Fig. 8).

Four putative species (e.g. BOLD:ADG3219 Fig. 6) were more abundant than others (Fig. 7) and thus could be potential target taxa for a functional screening approach. In contrast, a total of 202 specimens with BINs found in the Ptilodactylidae corresponds to a diversity of only five BINs, a total of 18 specimens with BINs placed as Latridiidae corresponds to only two BINs, as well as the only three specimens with BINs identified as Ciidae sharing the same BIN. It shows that all possible scenarios of relatively high and low abundance and diversity are present. When comparing the diversity found in the canopy with data from Malaise trap samples placed at the same sites (Cancian de Araujo et al. in prep.), it shows an obvious compartmentalisation between forest extracts with only 8.38% of BINs shared. A first attempt on understanding this complex diversity was submitted recently by Floren and collaborators as a paramount approach entitled "Integrative ecological and molecular analysis indicate high diversity and strict separation of canopy beetles in tropical mountain forests".

With this data release, we expect to highlight the diversity hidden in the canopy of the tropical forests, as well as support and encourage future robust analysis that will help to better understand the processes involved in such an impressive speciation.

The data released here are also an invitation to expert taxonomists to screen the species pages and contribute with further identification, where possible, based on the voucher images.

## Figures and Tables

**Table 1.** Number of trees per species and site of sampling

**Family**	**Species**	**Cikaniki**	**Gunung Botol**
Annonaceae	*Polyalthia subcordata*	2	
Elaeocarpaceae	*Sloanea sigun*	4	2
Fagaceae	*Litocarpus indutus*	6	3
Fagaceae	*Castanopsis javanica*	3	
Hamamelidiaceae	*Altingia excelsa*	1	
Lauraceae	*Litsea* sp.		2
Melastomataceae	*Memecylongar cinioides*	1	
Theaceae	*Schima wallichii*		4
